# Transverse Pericardial Sinus Hematoma From Type A Aortic Dissection: A Diagnostic Challenge

**DOI:** 10.7759/cureus.8322

**Published:** 2020-05-27

**Authors:** Raed Qarajeh, Kyle Lehenbauer, Ahmed Elkaryoni, Laith Derbas, Robert Tanenbaum

**Affiliations:** 1 Internal Medicine, University of Missouri Kansas City School of Medicine, Kansas City, USA; 2 Division of Cardiovascular Disease, Saint Luke's Mid America Heart Institute, Kansas City, USA; 3 Internal Medicine, University of Missouri, Kansas City, USA

**Keywords:** acute aortic syndrome, acute aortic dissection, type a aortic dissection, transverse pericardial sinus, pericardial hematoma

## Abstract

Ascending (type A) aortic dissection can rarely result in contained transverse pericardial sinus hematoma that compresses adjacent structures making diagnosis more challenging. We present a rare case of a 77-year-old man who presented with sudden-onset chest pain and was admitted for a presumed acute coronary syndrome. Coronary angiography did not show significant stenosis and ruled out acute coronary syndrome. Transthoracic echocardiogram showed extracardiac structure compressing on the left atrium; hence, we performed transesophageal echocardiogram which confirmed aortic dissection and revealed a hematoma in the transverse pericardial sinus. Intraoperatively, a large hematoma in the transverse pericardial sinus was extracted and revealed a posterior perforation of the ascending aorta that extended into the left atrium.

## Introduction

Acute aortic syndrome is a term used to describe a group of life-threatening aortic disorders that include classic aortic dissection [1]. Aortic dissection is relatively uncommon, but a catastrophic illness with poor prognosis. Ascending (type A) aortic dissection has a higher risk for life-threatening complications than descending aortic dissection; however, early diagnosis and treatment significantly improves survival [2,3]. It has been previously shown that in patients with type A aortic dissection, tamponade is less common if patients had prior cardiac surgery as they tend to develop contained hematoma [4]. In rare cases, aortic dissection can result in contained transverse pericardial sinus hematoma that compresses adjacent structures mimicking other diseases as left-sided heart failure or pulmonary embolism, therefore making diagnosis more challenging. 

## Case presentation

A 77-year-old man who underwent recent coronary artery bypass grafting (CABG) presented to the hospital with sudden-onset chest pain and dyspnea. On physical examination, he was normotensive with a normal heart rate. He had normal heart sounds without a murmur. Pulses were equal bilaterally. He had new bilateral lower limb edema.

The patient had a past medical history of coronary artery disease (CAD) status post remote percutaneous coronary intervention (PCI) and recent multi-vessel CABG one month prior to presentation. He also had hypertension, hyperlipidemia, tobacco use, and prostate cancer.

Our initial leading diagnosis was acute coronary syndrome from early graft occlusion. The remaining diagnoses on our differential included acute decompensated heart failure, acute aortic syndrome, acute aortic dissection, and pulmonary embolism.

**Figure 1:**
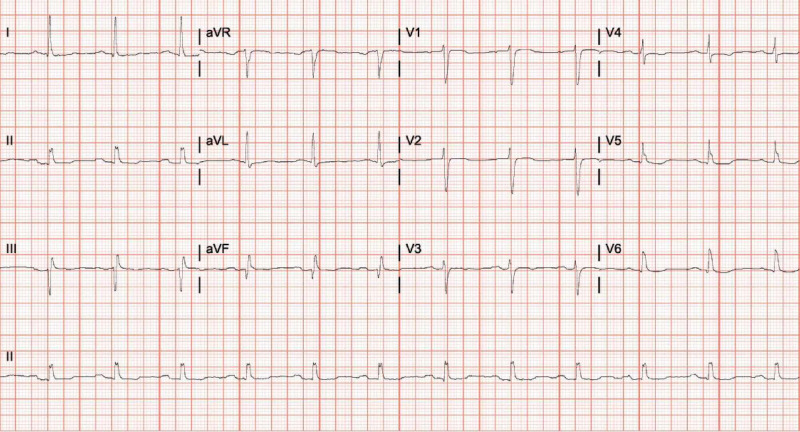
Electrocardiogram (EKG) EKG on presentation shows ST-segment depression in lateral leads.

**Figure 2:**
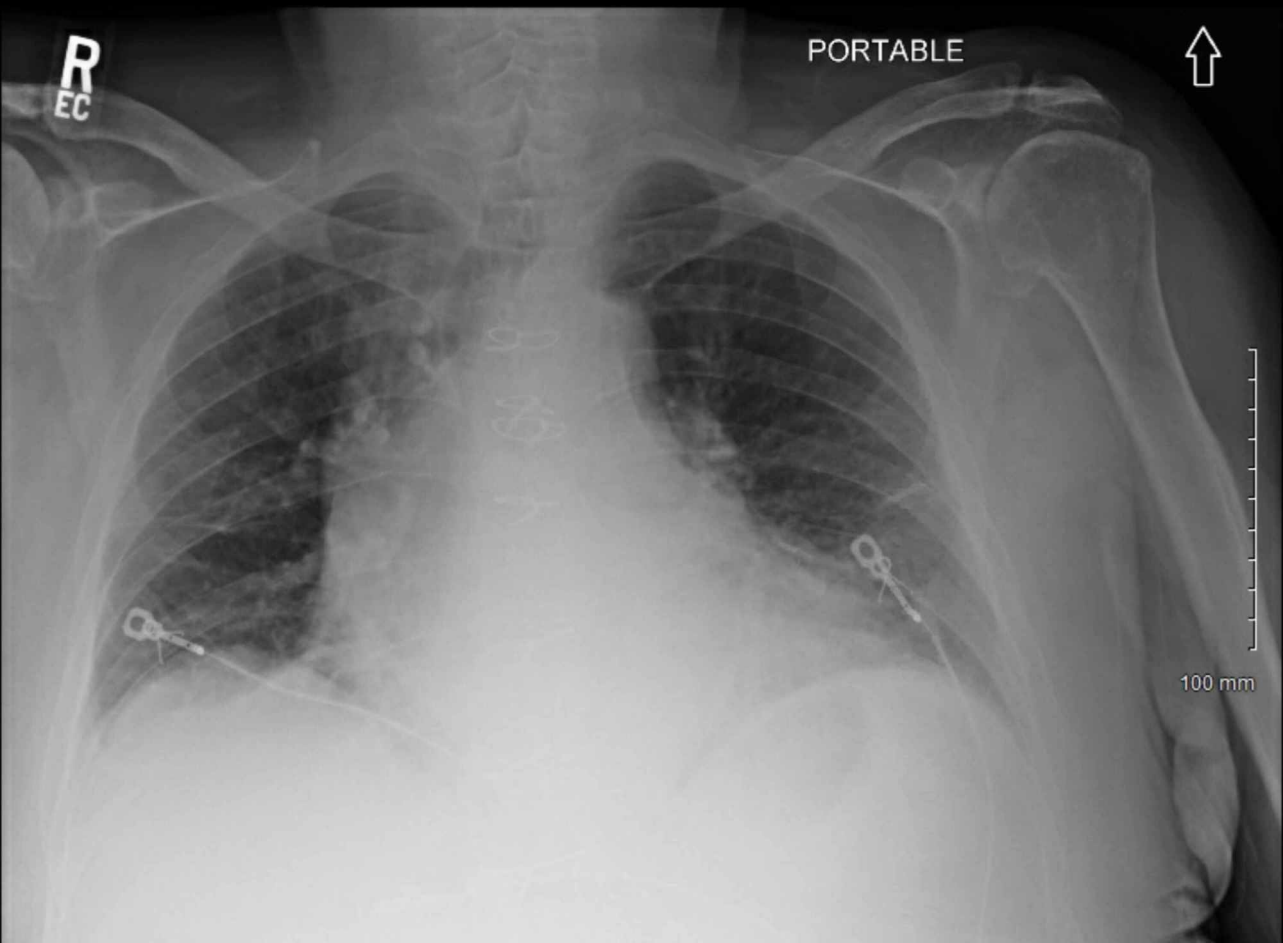
Chest x-ray Chest x-ray consistent with pulmonary edema and trace right pleural effusion.

Diagnostic studies revealed an elevated troponin value of 0.05 ng/ml that peaked at 8.83 ng/ml (normal range < 0.03), EKG with ST-segment depression in lateral leads (Figure 1), and chest x-ray consistent with pulmonary edema and trace right pleural effusion (Figure 2).

The patient was admitted for a presumed diagnosis of acute coronary syndrome (ACS) from early graft occlusion. He received nitroglycerin and dual antiplatelet therapy (DAPT), and was started on therapeutic anticoagulation with heparin. He received intravenous diuretic for pulmonary edema. The patient’s symptoms improved with these interventions. The following day, he underwent coronary angiography that revealed patent left internal mammary artery and saphenous vein grafts.

**Figure 3:**
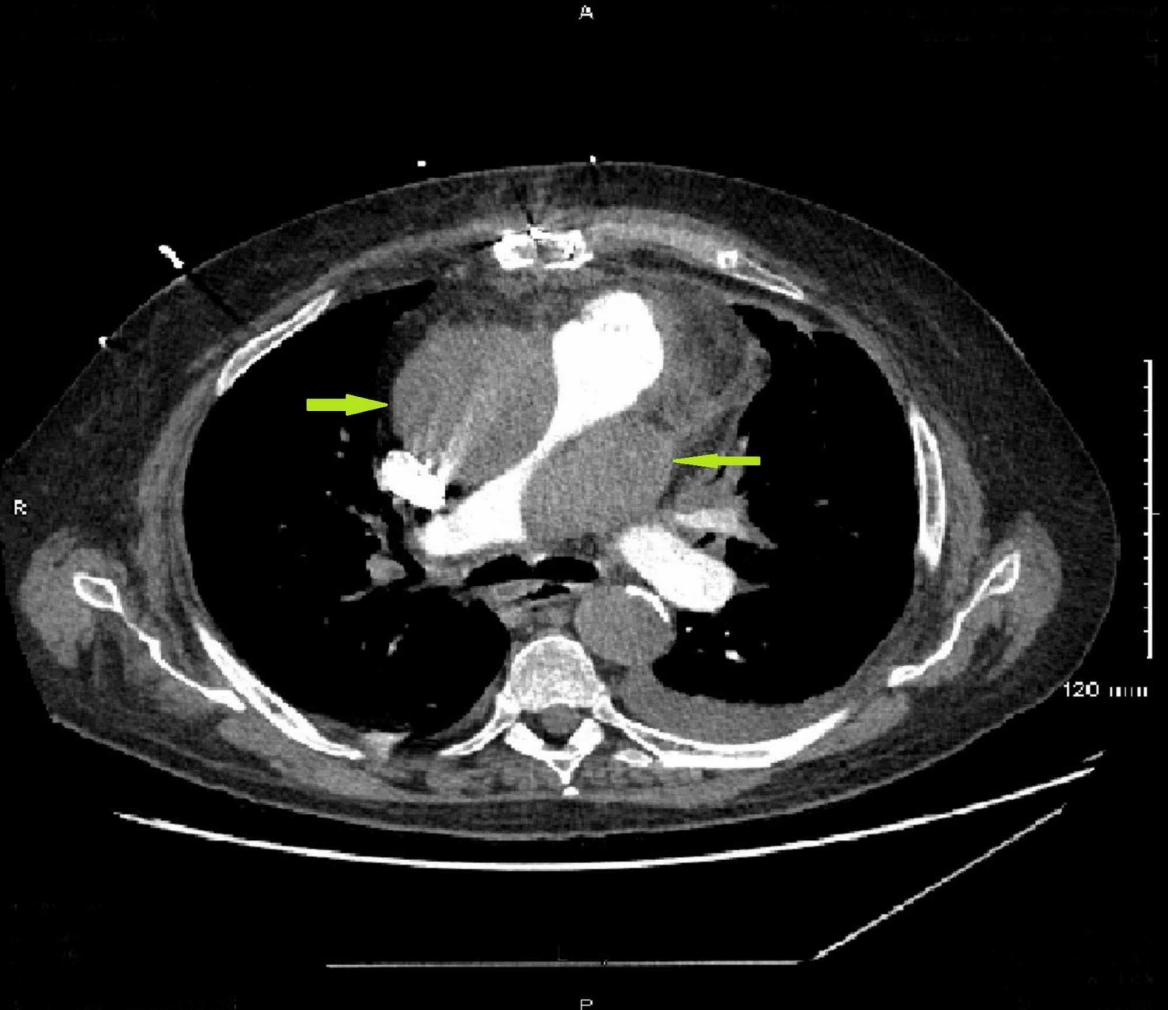
Computed tomography pulmonary angiogram (CTPA) CTPA without evidence of pulmonary embolism, however, shows a marked mass effect on the pulmonary trunk and right pulmonary artery (green arrows).

**Figure 4:**
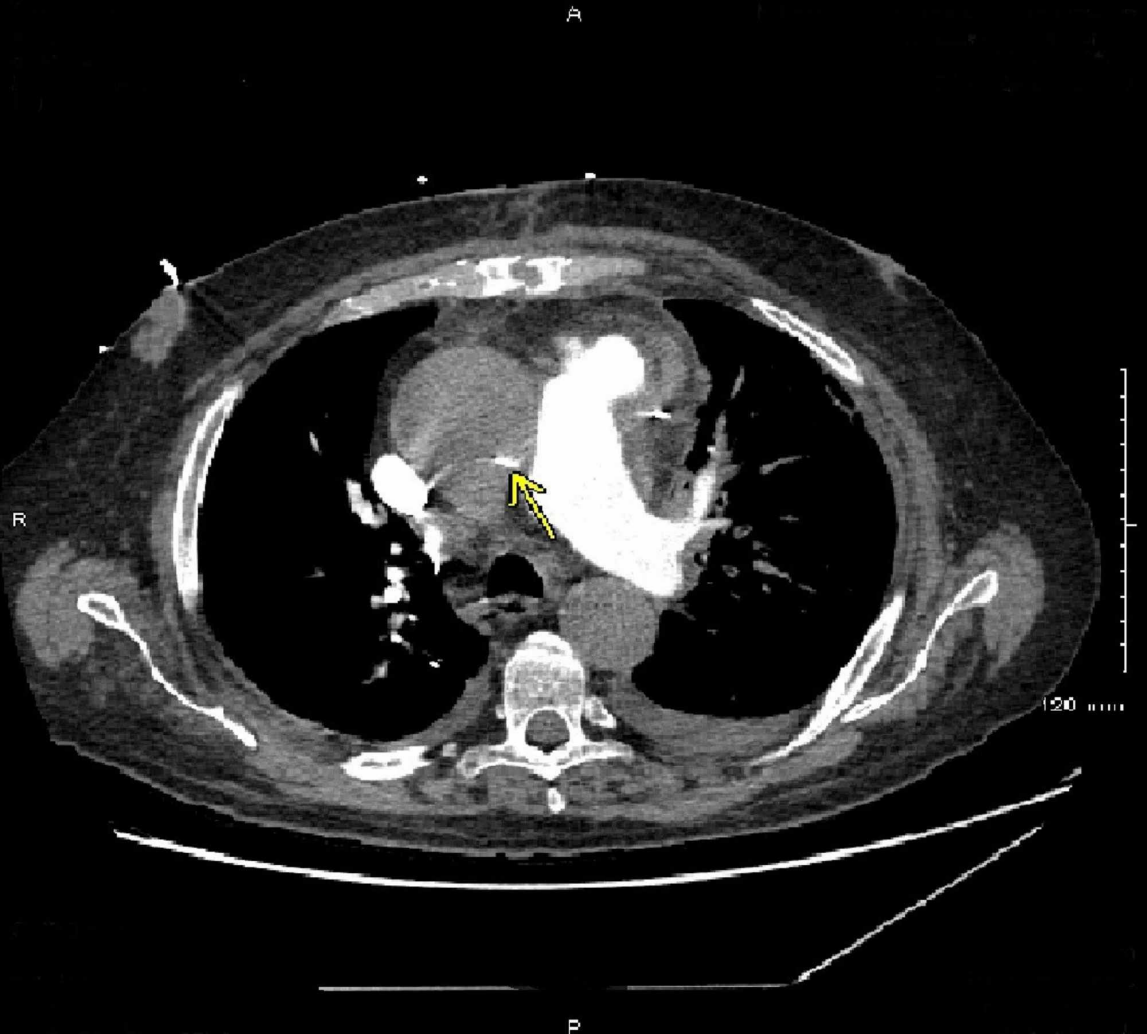
Computed tomography pulmonary angiogram (CTPA) concerning for aortic dissection CTPA shows a large ascending aortic aneurysm and displacement of intimal calcification (yellow arrow) concerning for Stanford type A aortic dissection.

Shortly after the catheterization procedure, the patient began to have worsening chest pain and shortness of breath. A transthoracic echocardiogram (TTE) was initially read as a technically difficult study but showed a normal left ventricular systolic function with an estimated ejection fraction of 65%, unchanged hypokinesis of the inferolateral walls (compared to prior TTE), and normal right ventricular size and systolic function. The patient then underwent a computed tomography pulmonary angiogram (CTPA) for further evaluation, which showed no evidence of pulmonary embolism; however, there was a marked mass effect on the pulmonary trunk and right pulmonary artery, and a large ascending aortic aneurysm with displacement of the intimal calcification concerning for Stanford type A aortic dissection (Figures 3, 4).

**Figure 5:**
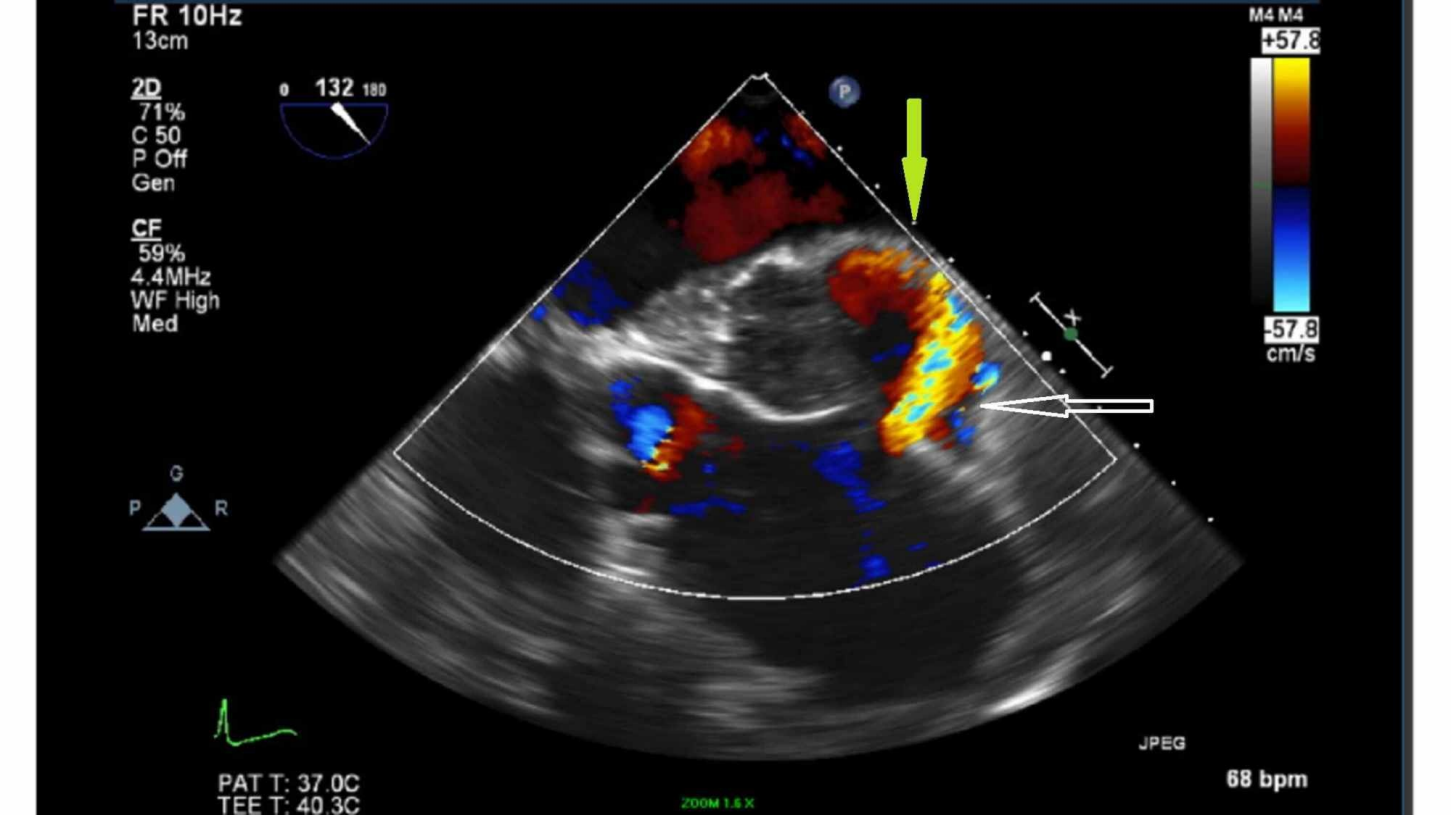
Transesophageal echocardiogram (TEE) TEE shows a proximal ascending aorta dissection, aortic wall tear, blood flow into the transverse pericardial sinus (white arrow), and a hematoma compressing the left atrium (green arrow).

**Figure 6:**
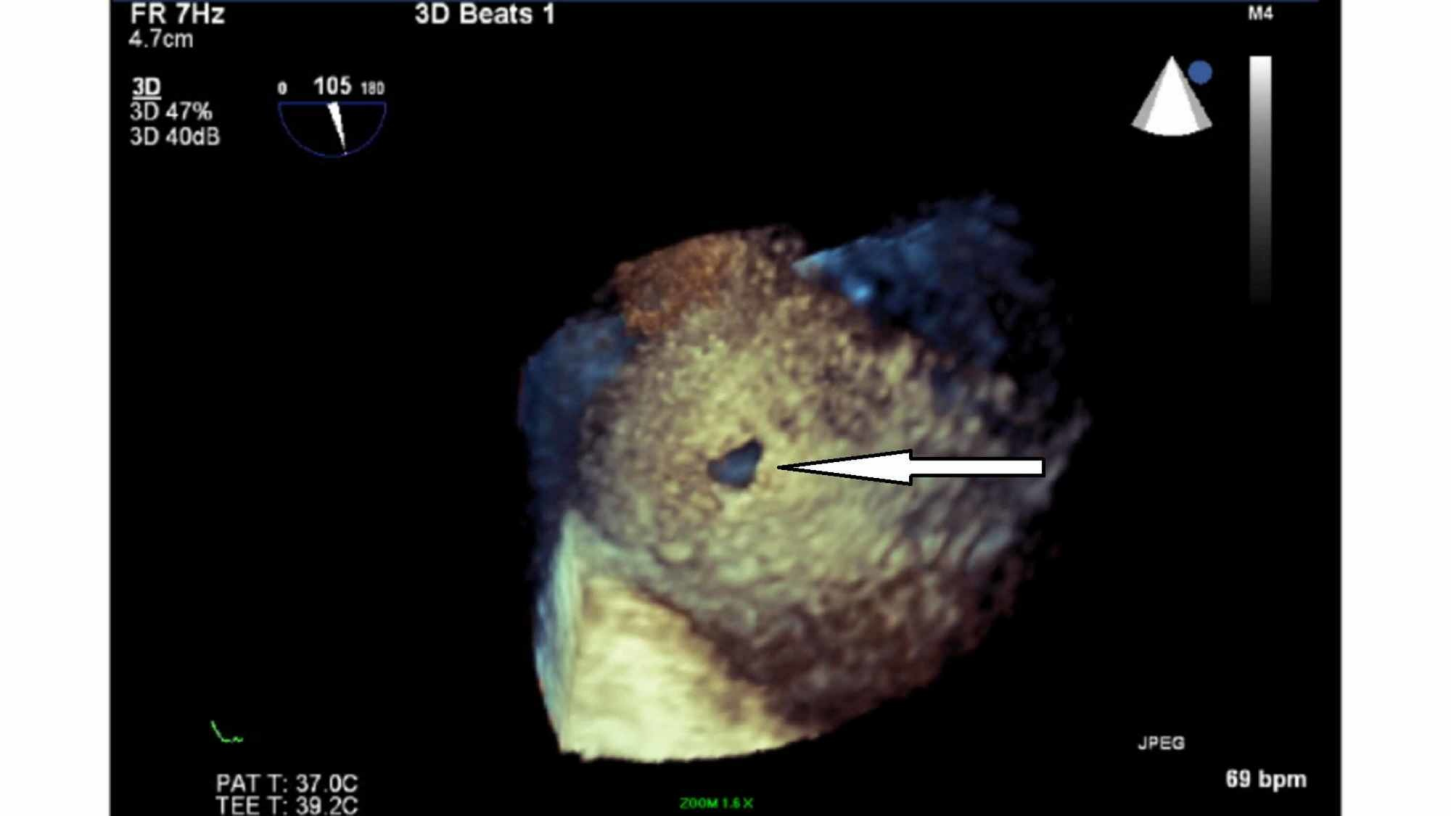
Real-time three-dimensional transesophageal echocardiography (RT-3D TEE) RT-3D TEE shows a posterior aortic tear (arrow).

As the patient’s respiratory status continued to deteriorate, he was transferred immediately to the intensive care unit for intubation. Blood pressure was controlled between 100 and 110 mmHg. A bedside transesophageal echocardiogram (TEE) revealed a proximal ascending aorta aneurysm and dissection. There was an aortic defect consistent with contained aortic rupture and flow into the transverse pericardial sinus. A hematoma in transverse pericardial sinus was compressing the left atrium (Figures 5, 6).

**Figure 7:**
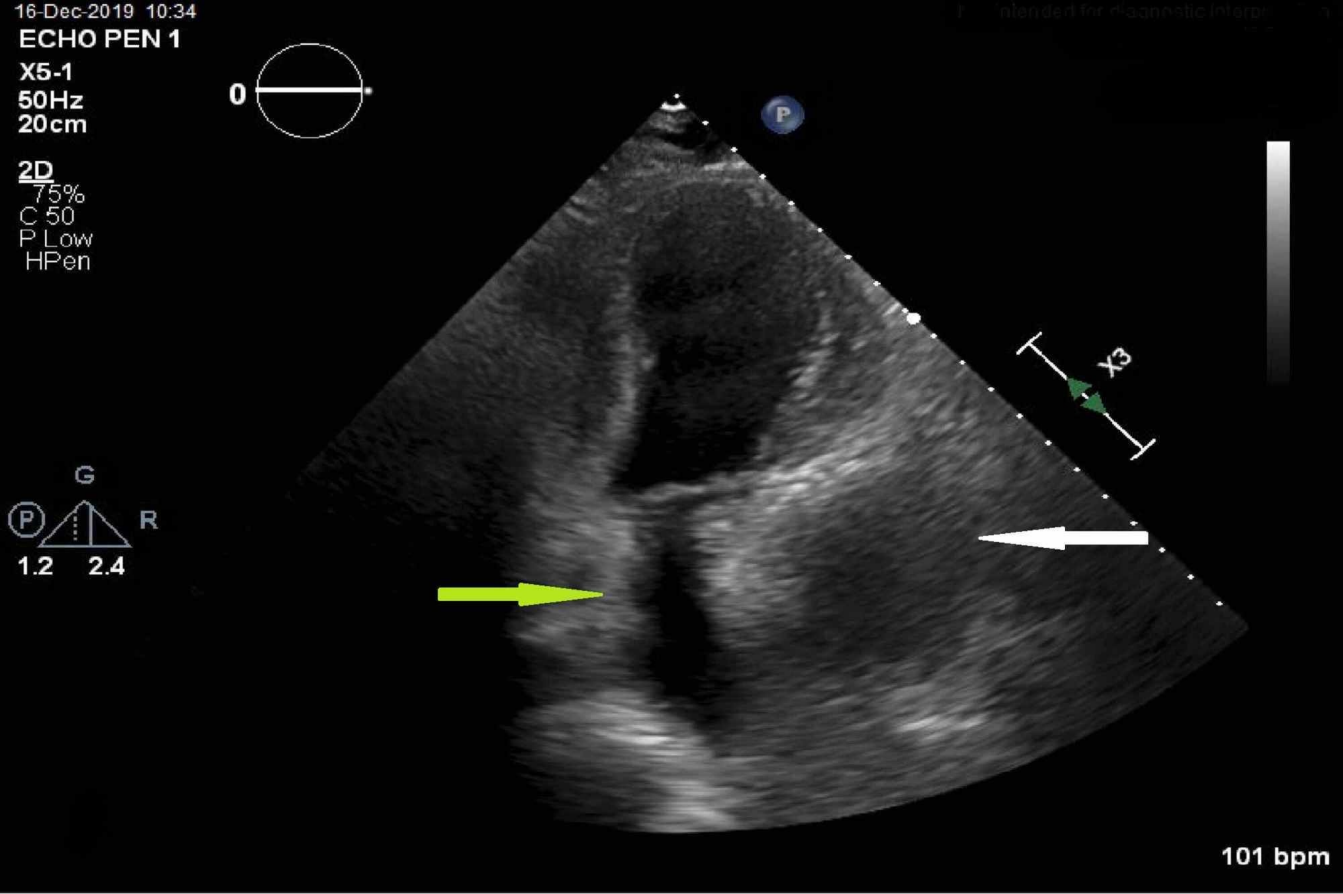
Transthoracic echocardiogram Transthoracic echocardiogram, apical two-chamber view, shows an extracardiac structure (white arrow) compressing the left atrium (green arrow) and mitral annulus.

Upon reviewing of the earlier TTE, there was a left atrial impression suggestive of an extracardiac structure compressing the left atrium and mitral annulus that was previously interpreted as an artifact (Figure 7).

The patient was taken urgently to the operating room. The dissection was found to extend only through the ascending aorta. A dissection flap extended to just below the innominate artery. Both vein grafts appeared to be intact without involvement in the flap. A large hematoma in the transverse pericardial sinus was extracted and revealed a posterior perforation of the ascending aorta that extended into the left atrium. The ascending aorta was repaired with a vascular graft. The vein grafts previously placed at his previous CABG surgery were reattached to the ascending graft. The left atrial tear was repaired. After separating the patient from bypass and instilling the heart with blood, the left atrial repair completely dehisced. Multiple attempts were made to repair this; however, the tissue was not viable enough to hold a suture. The patient had lost a significant amount of blood and, unfortunately, he died.

## Discussion

Acute aortic syndrome is a term used to describe a group of life-threatening aortic disorders that include classic aortic dissection, intramural thrombus, limited intimal tear, penetrating atherosclerotic aortic ulcer, and iatrogenic or traumatic dissection. Aortic dissection is relatively uncommon, but a catastrophic illness with poor prognosis. Mortality from aortic dissection can be secondary to cardiac tamponade, severe aortic insufficiency, or end-organ failure [1].

Ascending (type A) aortic dissection has a higher risk for life-threatening complications than descending aortic dissection; however, early diagnosis and treatment significantly improves survival [2]. Therefore, acute aortic dissection should be included in the differential diagnosis in patients presenting with chest pain even without specific physical findings. Troponin can be high in causes other than acute coronary syndrome; hence, in patients with chest pain and elevated troponin, especially with refractory symptoms and normal or unchanged/stable coronary anatomy, the index of suspicion for an alternative diagnosis like aortic dissection should be high. So far, there is no single biomarker to detect acute aortic dissection [3].

There are two pericardial sinuses: transverse and oblique. The transverse pericardial sinus is posterior to the pulmonary trunk and ascending aorta, superior to the left atrium, and anterior to the superior vena cava. Our patient had an aortic dissection resulting in a contained transverse pericardial sinus hematoma, most likely due to post-surgical adhesions related to his recent CABG surgery. It has been shown that tamponade is less common in patients with prior cardiac surgery as they tend to develop a contained hematoma [4]. Aortic dissection with contained hematoma makes diagnosis more challenging since a hematoma can compress adjacent structures mimicking other diseases such as left-sided heart failure or pulmonary embolism. In this case, a hematoma was compressing the left atrium and pulmonary artery causing severe dyspnea and pulmonary edema.

The 2010 U.S. guideline for thoracic aorta disease has recommended CT to evaluate for suspected aortic dissection [5]. It also reported that TEE is superior to TTE in the assessment of the thoracic aorta. Major advantages of TEE over other imaging modalities include the ability to perform bedside patient evaluation, rapid imaging time, and lack of intravenous contrast or ionizing radiation. In addition, TEE can rapidly provide information related to aortic dissection complications, such as cardiac tamponade, aortic regurgitation, and proximal coronary artery involvement. In our patient, bedside TEE helped in the identification of aortic dissection, hematoma, as well as blood flow in the transverse sinus and ruled out tamponade or aortic insufficiency.

A left atrial impression seen on TTE could be a sign of extracardiac pathology compressing on the left atrium. A differential of left atrial mass effect from extracardiac structure includes gastrointestinal etiologies, pulmonary etiologies, mediastinal structures, or a pericardial hematoma [6]. Unfortunately, this feature was initially missed on the TTE read which delayed definitive diagnosis.

Finally, a high degree of suspicion and early diagnosis are essential to successfully manage this surgical emergency and to avoid inappropriate treatment that might increase the risk of surgery-related complications. Unfortunately, our patient was loaded with ticagrelor on presentation for presumed ACS which contributed to exsanguination. Studies have shown that the use of DAPT prior to acute type A aortic dissection repair was associated with a higher risk of bleeding and transfusions, but not associated with mortality. However, major bleeding was higher in patients who received DAPT and major bleeding per se was associated with higher mortality [7].

## Conclusions

Clinicians should have a high index of suspicion for acute aortic dissection in patients presenting with chest pain even without classic dissection clinical characteristics or physical findings. Left atrial impression on TTE can be a sign of pericardial hematoma. Ascending (type A) aortic dissection can result in contained transverse pericardial sinus hematoma making diagnosis more challenging.
